# Strongyloides colitis, a rare but important mimic of Crohn’s disease, resulting in coma and multi-organ failure: a case report

**DOI:** 10.1186/s40792-022-01568-6

**Published:** 2022-11-30

**Authors:** Sabah Uddin Saqib, Sumit Sood, Ling Wong, Abhilasha Patel

**Affiliations:** University Hospital Coventry and Warwickshire Trust, Coventry, UK

**Keywords:** Strongyloides colitis, Strongyloidiasis, Crohn’s, Tropical infection, Hyperinfection syndrome

## Abstract

**Background:**

Strongyloides colitis is a severe form of strongyloidiasis that carries a high mortality rate if untreated. There is an overlapping clinical presentation between Strongyloides colitis and Crohn’s disease. Here, we present a case of a patient who was diagnosed with Crohn’s disease and was treated with immunosuppressant therapy which resulted in a poor outcome.

**Case presentation:**

A middle-aged, native African male presented with diarrhea, abdominal pain, and weight loss. Colonoscopy showed some patchy inflammation in the caecum, which on biopsy was suggestive of Crohn’s disease. He had a short course of steroids before being admitted to an emergency with abdominal pain, diarrhea, malnutrition, and severe weight loss. Initial conservative treatment failed, and he became acutely unwell and septic with peritonitis. Laparotomy was carried out, which showed mild inflammation in the terminal ileum, which was not resected. Postoperatively, the patient became comatose and went into multi-organ dysfunction. He failed to progress, and a further laparotomy and subtotal colectomy were performed on the 12th postoperative day. His multi-organ failure progressed, and he succumbed to death 4 days later.

**Discussion:**

*Strongyloides stercoralis* is a parasite causing an enteric infection in animals and humans. Strongyloidiasis in immunocompetent individuals is usually an indolent disease. However, in immunocompromised individuals, it can cause hyperinfective syndrome. Patients with strongyloid colitis should undergo colonoscopy and biopsy where acute inflammation with eosinophilic infiltrates indicates parasitic infiltration of the colonic wall. Surgery is generally not indicated, and any surgical intervention with misdiagnosis of a flare-up of IBD can be very detrimental to the patient.

**Conclusion:**

Strongyloid colitis can very harmfully mimic Crohn’s colitis, and the use of steroids and immunosuppressants can disseminate parasitic infection. Hyperinfection syndrome can lead to sepsis, organ dysfunction, and comma. Disseminated infection carries a high mortality.

## Background

Strongyloides colitis is a severe form of strongyloidiasis that carries a high mortality rate if untreated [1]. Human pathogenic parasitic roundworms cause the disease, and human autoinfection is very common. The condition is endemic in tropical and subtropical countries, Africa, and South America [2]. There is overlapping clinical presentation and histomorphology between Strongyloides colitis and inflammatory bowel disease [3]. Therefore, a low index of suspicion can lead to misdiagnosis and fatal consequences. This is mainly due to the use of steroids and immunosuppressants to treat the condition as a flare-up of Crohn’s, which results in systemic manifestations of parasitic infection, which leads to high morbidity and mortality. However, if correctly recognized, Strongyloides colitis is an easily treatable condition in immunocompetent people using antihelminthic agents like ivermectin or albendazole. Here, we present a case of a patient who was diagnosed with Crohn’s disease and was treated with immunosuppressant therapy. His clinical condition rapidly deteriorated, and despite surgical treatment, he developed multi-organ failure and succumbed to the illness.

## Case presentation

**Fig. 1:**
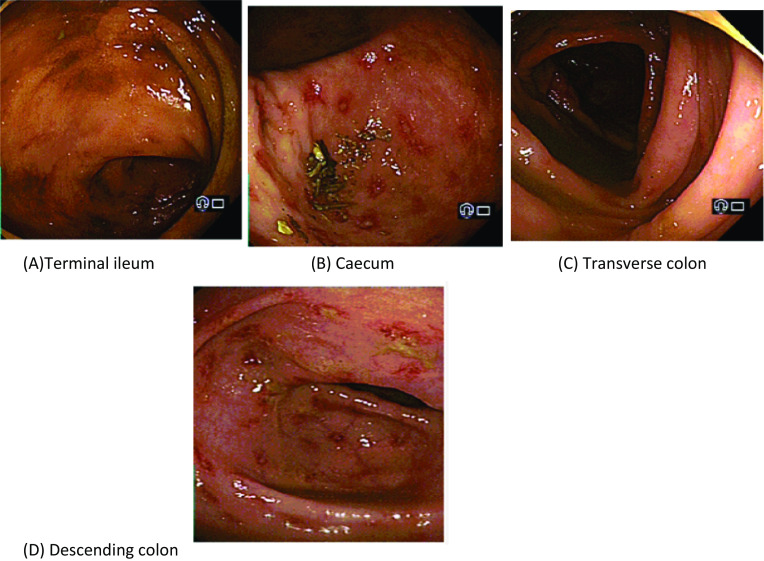
Shows colonoscopy findings in different parts of colon **A** to **D**

A middle-aged male of African ethnicity with Type II diabetes mellitus presented to the colorectal outpatient clinic with a 1-month history of bloody diarrhea, abdominal pain, and weight loss. He underwent a colonoscopy, which showed some patchy moderate erythema with multiple scattered small ulcers in the terminal ileum, caecum, transverse, and descending colon (Fig. 1). Biopsy of these areas showed cryptitis and ulceration. Within the lamina propria, there was moderate chronic inflammation with notable eosinophils. This raised possibility of Crohn’s disease or possible infectious etiology. He was initially treated with steroids, but his symptoms progressively worsened to the extent that he was admitted as an emergency with worsening abdominal pain and 30 kg weight loss in the preceding 3 months.

**Fig. 2:**
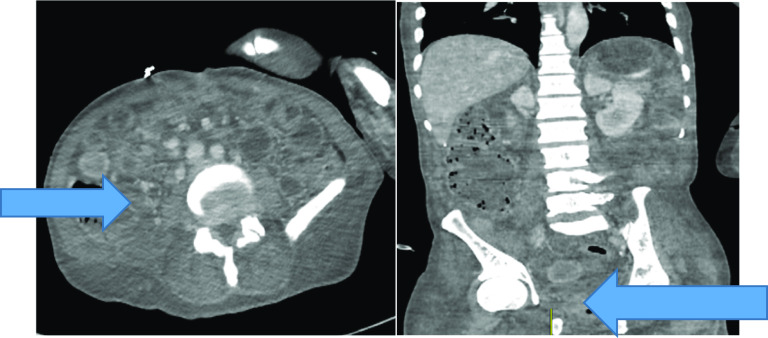
Shows axial and coronal sections of CECT (porto-venous phase) in which the blue arrow points towards the non-enhanced terminal ileum

During this emergency hospital admission, he had extensive investigations. His hematological and biochemical workup showed Hb of 117 g/L, WBC of 14.38 × 10^9^/L, CRP of 44 mg/L, and creatinine of 70 umol/L. Stool microscopic examination was negative for any bacterial growth or presence of larvae. Initial CT abdomen with intravenous contrast showed inflammation in the terminal ileum and caecum. His case was discussed in the inflammatory bowel disease multidisciplinary team meeting (IBD-MDT), and a flare-up of Crohn’s was established as the best possible diagnosis. He was given intravenous steroids followed by infliximab therapy. Total parenteral nutrition was instituted to support his nutritional intake. His symptoms did not improve, and he was then counseled for ileocolic resection and stoma formation. Initially, the patient was adamant that he did not want surgery because of the risk of having a stoma formation. His condition progressively deteriorated, and on the 3rd week of admission, he developed generalized peritonitis, and a contrast-enhanced CT (CECT) scan (porto-venous phase) raised suspicion of terminal ileum necrosis (Fig. 2).

He was taken to the theater as an emergency, and at operation, mild inflammation was noted in the terminal ileum with no evidence of ischemia. Therefore, bowel resection was not performed, and he was admitted to the intensive care unit. His neurological condition declined after surgery. A subsequent CT scan of his head and MRI Brain did not show any organic cause for his low level of consciousness. On the 10th postoperative day, his EEG showed generalized low amplitude slowing of cerebral activity in the form of predominately delta waves, suggestive of a severe degree of cerebral dysfunction of nonspecific etiology. His ammonia level was normal (29 umol/L), while CSF cultures showed moderated growth of Enterococcus. He developed further rectal bleeding, and his hemoglobin dropped to 69 g/L. His sepsis continued to worsen, and encephalopathy persisted. At this stage, CT chest abdomen, and pelvis with intravenous contrast was performed, which again showed inflammation around the area of the terminal ileum.

Following the extensive discussion with the local IBD-MDT and critical care specialist, a decision was made for re-laparotomy. The intraoperative findings showed mild inflammation in the terminal ileum and caecum. He underwent a subtotal colectomy with ileostomy. Despite maximal medical support, he succumbed on the 6th postoperative day.

**Fig. 3:**
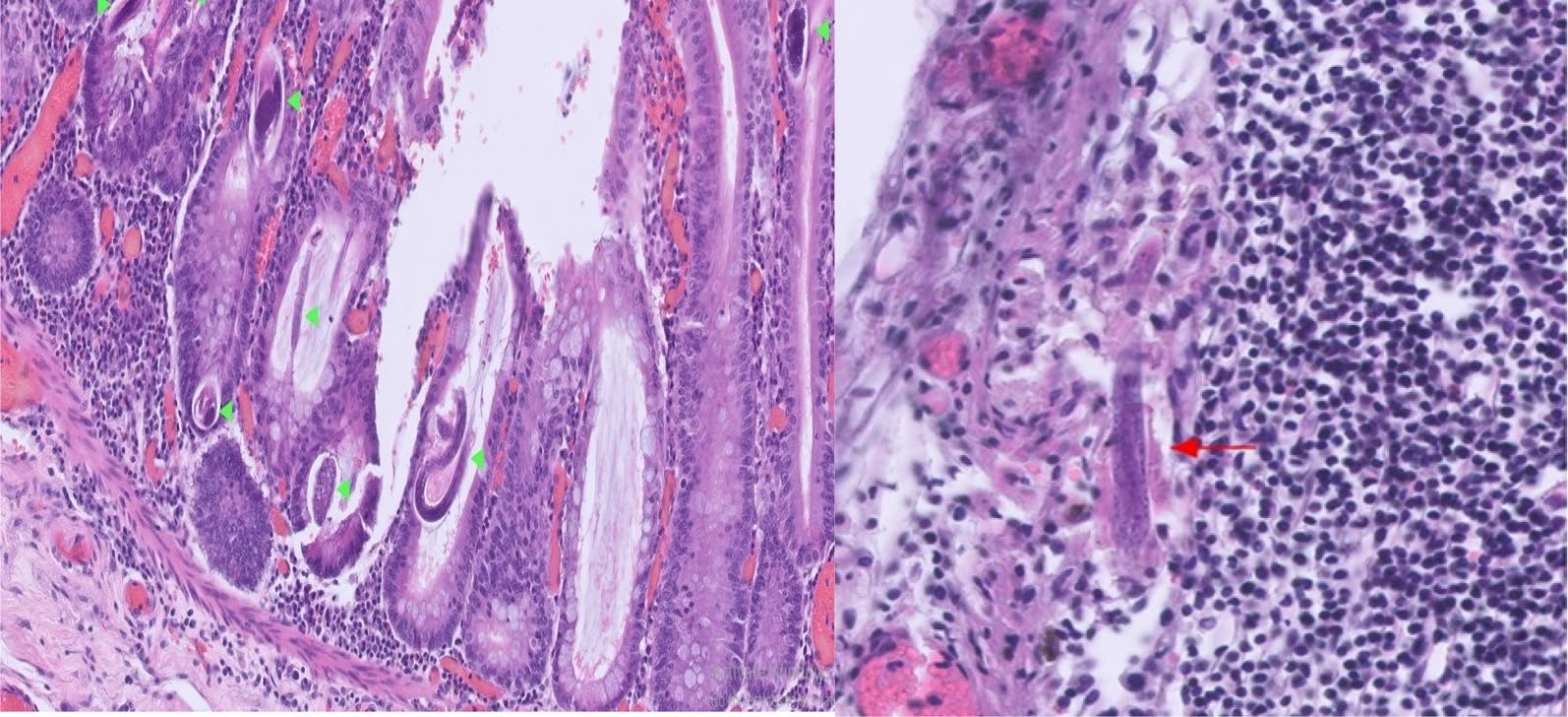
Shows green arrowhead indicating strongyloids in bowel lumen and red arrow shows strongyloid inside lymph node

Histopathological analysis of colon and terminal ileum showed parasite/worm-like organisms (Strongyloides) in the bowel wall and lymph nodes with inflammatory changes secondary to parasite infection (Fig. 3). The final report of histopathology did not support the diagnosis of Crohn’s disease. His slides were sent to the London Institute of tropical Illness for notification and further investigations. This confirmed the diagnosis of *Strongyloides stercoralis*.

## Discussion

*Strongyloides stercoralis* (*S. stercoralis*) is a parasite causing an enteric infection in animals and humans. It is primarily endemic in the tropics and subtropics, with a prevalence of over 70% in selected countries, affecting up to 370 million humans worldwide [4, 5]. Increased migration and travel to the United Kingdom and Europe from these countries have led to a rise in cases in the recent past [5, 6]. The disease is transmitted through a nematode and has the potential to cause autoinfection in the human host. Larvae enter through skin penetration from soil polluted with the parasite and reach the lungs through circulation. Later humans ingest these larvae, and then they get to the intestine [7, 8]. Only females reproduce in the intestine to reach adulthood. The eggs hatch in the intestine and young larvae are then excreted in the feces. It takes about 2 weeks to reach egg development from the initial skin penetration.

Strongyloidiasis in immunocompetent individuals is usually an indolent disease. However, in immunocompromised individuals, it can cause a hyperinfective syndrome [9, 10], also called disseminated strongyloidiasis, due to the reproductive capacity of the parasite inside the host. This hyperinfective syndrome can have a mortality rate close to 90% if disseminated. The disease can cause symptoms of colitis which can harmfully mimic inflammatory bowel disease (IBD) [11]. As its clinical presentation and histomorphology overlap with IBD, the disease can be mistakenly treated with steroids and immunosuppressants, which increases the chances of hyperinfection syndrome and systemic shutdown. A very high index of suspicion is required to differentiate strongyloide stercoralis from Crohn’s disease as the former is treated with antihelminthic agents like ivermectin and albendazole [12]. Immunosuppressants are strongly contraindicated as strongyloidiasis can disseminate with their use.

Diagnosis of strongyloide stercoralis is challenging to establish, and stool samples can confirm the presence of this parasite. Other techniques used include direct fecal smears, culturing fecal samples on agar plates, and serodiagnosis through ELISA. However, diagnosis can be difficult because of the day-to-day variation in juvenile parasite load [13, 14]. Patients with strongyloide colitis should undergo colonoscopy and biopsy where acute inflammation with eosinophilic infiltrates indicates parasitic infiltration of the colonic wall. A biopsy may show larval forms, especially in patients living or traveling from endemic areas [15, 16].

The disease is generally asymptomatic in immunocompetent patients but can be disseminated dreadfully in an immunocompromised patient or after initiation of treatment reserved for IBD. In disseminated strongyloidiasis, bacteremia may develop due to translocation of enteric bacteria through the tract created by invading filariform larvae, or bacteria itself can be carried on the larva. Systemic sepsis and septic shock are inevitable if the disease is widely disseminated. Cardiovascular, pulmonary, and renal shutdown occurs along with altered neurological status, and likewise, in our case, the patient can go into a coma because of septic encephalitis. Surgery is generally not indicated, and any surgical intervention with misdiagnosis of a flare-up of IBD can be very detrimental to the patient as it can increase catabolic stress and deplete the physiological reserves of the patient. If early diagnosis is not established, this vicious cycle leads to mortality, as presented in our case.

## Conclusion

Strongyloid colitis can very harmfully mimic Crohn’s colitis, and the use of steroids and immunosuppressants can disseminate parasitic infection. Hyperinfection syndrome can lead to sepsis, organ dysfunction, and comma. Disseminated infection carries a high mortality.

## Data Availability

All data will be available on request, keeping the anonymity of the patient.

## Abbreviations

CRP: C-reactive protein
CSF: Cerebrospinal fluid
CT: Computed tomography
CECT: Contrast-enhanced CT scan
EEG: Electroencephalogram
ELISA: Enzyme-linked immunoassay
Hb: Hemoglobin
MDT: Multidisciplinary team
IBD: Inflammatory bowel disease
MRI: Magnetic resonance image
WBC: White blood cell count
